# Functional performance and symptomatology of adults with skeletal dysplasia across self-care, mobility, and cognition—a cross-sectional study

**DOI:** 10.1093/jbmrpl/ziaf008

**Published:** 2025-01-12

**Authors:** Daphne Nguyen, Penelope Ireland, Verity Pacey

**Affiliations:** Department of Health Sciences, Faculty of Medicine, Health and Human Sciences, Macquarie University, Sydney, NSW 2113, Australia; Queensland Paediatric Rehabilitation Service, Queensland Children’s Hospital, South Brisbane, QLD 4101, Australia; Department of Health Sciences, Faculty of Medicine, Health and Human Sciences, Macquarie University, Sydney, NSW 2113, Australia

**Keywords:** skeletal dysplasia, achondroplasia, functional independence, self-care, mobility, cognition

## Abstract

Individuals with skeletal dysplasia (SD) experience physical challenges in performing everyday activities as a result of their altered biomechanics and systemic comorbidities. The purpose of this study was to objectively assess functional performance and identify symptomatology across self-care, mobility, and cognitive tasks among adults with SD. The secondary aim was to describe any differences in performance between individuals with proportionate forms of SD compared with those with disproportionate forms. A cross-sectional online survey and functional assessment, the Functional Independence Measure (FIM), was administered to adults diagnosed with SD. Summary statistics were computed and analyzed. Survey open-ended text responses were examined. Data were collected on 40 adults (median age 41.2 yr) presenting with 10 types of SD (14 with proportionate SD, 26 disproportionate SD). A total of 39 (97.5%) participants required assistance or modification when completing functional activities. Increased reliance on assistance was observed across self-care and mobility tasks (median FIM scores 51/56 and 31.5/35 respectively), compared with cognitive items (median FIM score 34.5/35). Up to 50% of participants reported experiencing pain, fatigue, and/or other symptoms during self-care and mobility tasks. These symptoms were more prevalent when completing self-care activities at home and when mobilizing in the community. Participants with proportionate forms of SD demonstrated higher levels of independence in upper body dressing, toileting, and bed/chair/wheelchair transfers, whereas participants with disproportionate forms had higher scores in eating and grooming tasks. Adults with SD demonstrated functional challenges and symptomatology associated with activities of daily living. Overall, there are minimal differences in FIM scores between individuals with proportionate forms of SD and those with disproportionate forms. Use of assistive equipment, modifications, and allowing extra time with tasks may improve independence among this population group.

## Graphical Abstract

**Graphical Abstract:**
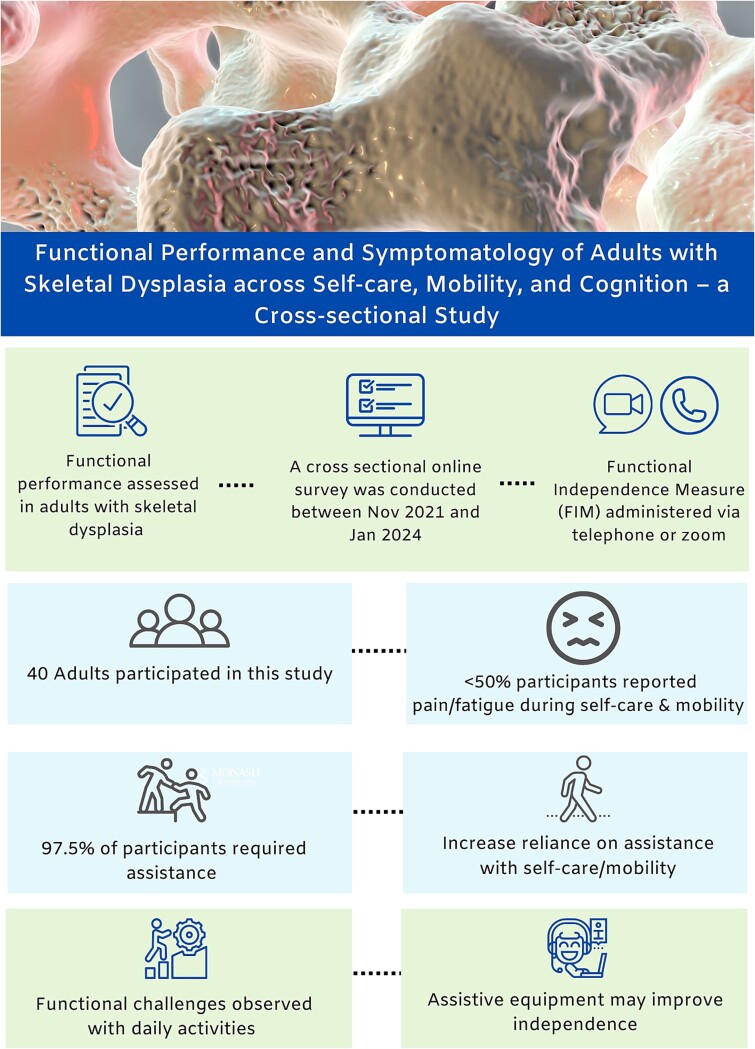

## Introduction

Skeletal dysplasia is a term that encompasses a heterogeneous group of over 450 congenital disorders affecting approximately 1 in 5000 births, and present with abnormalities in the growth and development of bone and cartilage.^1^^,^^2^ These conditions are frequently characterized by marked short stature and can be further classified as proportionate or disproportionate based on the presentation of trunk-to-limb length ratio.^2^ Individuals with skeletal dysplasia present with various secondary impairments, which may affect cardiorespiratory, musculoskeletal, neurological, and ears, nose, and throat systems.^2–7^ Common impairments observed among adults include thoracolumbar kyphosis, obesity, spinal stenosis, reduced exercise tolerance, and pain.^2–7^ Other complications may include hearing loss and cognitive developmental delays.^7^ The clinical features and impairments associated with skeletal dysplasias and their subsequent complications may impact on mobility, self-care, and cognitive functioning, and result in activity limitations and participation restrictions.

There are limited studies evaluating the functional performance of adults with skeletal dysplasia. Previous research has primarily focused on individuals with disproportionate conditions, most commonly achondroplasia, and has identified that adults with skeletal dysplasia experience greater limitations in their physical functioning compared with average-statured peers.^4^^,^^5^^,^^8–10^ Challenges with mobilizing, reaching, and performing self-care and household tasks independently are frequently reported, which is likely due to anatomical variations and impairments associated with individual forms of skeletal dysplasia.^4^^,^^6^^,^^8–11^ Individuals frequently require use of more assistive equipment, environmental modifications, or help from others to complete daily tasks.^6^^,^^9^^,^^11^ While studies have identified physical challenges in completion of self-care tasks, the levels and types of assistance required to perform these daily activities and variations in performance between individuals with proportionate and disproportionate forms of skeletal dysplasia have not been quantified.

Pain has been identified as a prevalent symptom among most individuals with skeletal dysplasia, which impacts on quality of life.^3–9^^,^^12^ A study by Alade et al.^10^ identified that approximately 70% of participants with skeletal dysplasia reported experiencing frequent pain, ranging from mild to severe, which interfered with everyday activities. While this study also examined physical function within the cohort, potential links between pain and individual daily self-care activities or tasks were not investigated.^10^ Furthermore, recent research exploring symptomology related to function in individuals with skeletal dysplasia has primarily focused on pain and have not yet aimed to identify or describe other symptoms that may be associated with function.

When considering assessment of function in individuals with skeletal dysplasia, it is important to quantify both level and type of assistance that is required. The Functional Independence Measure (FIM) is a standardized instrument developed to quantify type and amount of assistance individuals with a disability may require to complete self-care, mobility, and cognitive tasks.^13^ It can be used to evaluate independence levels and carer burden in any adult population where functional impairments exist.^13^ The FIM has been reported to have good psychometric properties across a wide range of patient populations, raters, and settings, including remote administration.^13^^,^^14^ Previous research has demonstrated FIM to be reliable in measuring functional independence in adults with skeletal dysplasia.^4^ It has been utilized in adult populations with mucopolysaccharidoses,^15^^,^^16^ and the pediatric version, WeeFIM-II, has been used in children with achondroplasia.^17^

This study aimed to (1) quantify the range of functional performance and identify symptomatology in adults with skeletal dysplasia across self-care, mobility, and cognition domains, and (2) identify and describe differences in functional performance between individuals with proportionate and disproportionate forms of skeletal dysplasia.

## Materials and methods

### Study design

A cross-sectional study, based in Australia, utilizing survey data and formal functional assessment of adults with skeletal dysplasia was conducted. Ethical approval was granted by the Macquarie University Human Resource Ethics Committee (#10717).

### Participants

Participants were recruited between November 2021 and January 2024 in collaboration with the Short Stature People’s Association of Australia (SSPA) and the Osteogenesis Imperfecta Society of Australia advocacy groups. Information on the study was provided through SSPA social media advertisements, newsletters, and conferences.

Participants were eligible for inclusion if (1) they were aged 18 or older, and (2) had a clinical diagnosis of a form of skeletal dysplasia. They were excluded if they were unable to provide informed consent, communicate effectively in English, and/or had an additional musculoskeletal or neurological condition not associated with skeletal dysplasia, which impacted their ability to complete self-care, mobility, or cognitive tasks.

### Measures

The FIM was used to assess performance in everyday activities among adult participants with a skeletal dysplasia. The FIM assesses 13 skill motor areas across self-care and mobility, in addition to 5 skill cognitive areas, and is designed to reflect typical performance in a usual environment.^13^ Each item is scored on an ordinal scale from 1 to 7, providing a total score between 18 and 126, with higher scores indicating greater levels of independence.^13^ A score of 7 indicates that the person is completely independent with the task, whereas a score of 6 demonstrates modified independence, signifying the person can complete the task without physical assistance but may require extra time or adaptive equipment to support them.^13^ A score of 5 indicates the person requires a helper to assist with the setup of the task and/or providing hands-off supervision or curing.^13^ Scores between 1 and 4 specify the person requires varying levels (minimal, moderate, maximal, total) of physical hands-on assistance from another person to complete the task.^13^

### Procedure

Eligible participants were invited to complete an online survey that collected demographic data and information on functional performance and participation. Data regarding diagnosis, age, gender, additional co-morbidities, and occupation were recorded through tick box and open-ended responses. Survey questions explored topics regarding access to government-level disability support (named the National Disability Insurance Scheme in Australia), use of healthcare professionals, community accessibility, mobility, self-care, leisure activities, public and private transportation, and communication. The survey format included multiple choice checkboxes, short open text, slider scales, and drop-down list options. Additionally, there were optional longer open-ended text questions for participants to elaborate on their answers if they so choose, or to discuss other areas of functional challenges in daily activities not yet addressed in the survey.

Consenting participants were contacted to complete the FIM assessment, via telephone or Zoom, administered by a formally trained team member accredited through the Australasian Rehabilitation Outcomes Centre. Descriptive notes were taken during the FIM assessment on patient-reported data, which correlated with their scoring, for example, types of assistive equipment and modifications used. Additionally, during this interaction, assessors collected participants’ scores on The Screening Tool for Everyday Mobility and Symptoms (STEMS), to be used as a demographic factor. The STEMS is an instrument developed for individuals with skeletal dysplasia that assesses the use of mobility aids and symptomatology associated with mobility across home, school/work, and community settings.^18^ Based on the STEMS, participants receive a score between 1 and 5 dependent on whether they required a mobility aid, and what kind of aid was required for each environment (1—independent on all surfaces including stairs, 2—use of stick(s), 3—use of crutches, 4—use of wheeled walking device, and 5—use of wheelchair or mobility scooter).^18^ They also receive an alphabetical score based on pain and/or fatigue that they experience when mobilizing in each environment (A—no pain/fatigue, B1—pain only, B2—fatigue only, and C—both pain and fatigue).^18^

Following completion of the FIM assessments, participants received an emailed copy of a formal report with their individual FIM results. Those with incomplete surveys were followed up through email and/or phone where possible.

### Data analysis

All statistical analyses were conducted using Microsoft Office Excel version 2204. Demographic data were described through standard descriptive statistics. Due to the small sample size and non-normal distribution of scores, medians were used to denote central tendency. Continuous data were recorded as medians and ranges, while categorical data were documented as frequencies and percentages.

Functional performance was described using the FIM data. Medians and ranges were computed for each of the 18 FIM items, domain subtotals, and overall FIM scores. The percentage of total participants scoring independent (complete and modified), supervision, or hands-on assistance (minimal, moderate, maximal, total) scores across each item was recorded.

To evaluate differences between participants with proportionate and disproportionate forms of skeletal dysplasia, participants were categorized based on the trunk-to-limbs length ratio associated with their condition (Table 1).^1^^,^^2^^,^^21–27^ As a result of the small sample size, statistical tests could not be computed with acceptable statistical power. Therefore, differences between the 2 participant groups were evaluated using descriptive statistics. The median scores and ranges for each item, subtotals, and total FIM score were reported for each participant category. Data from the open-ended text survey questions were categorized by DN into the following groups: (1) avoidance of activities, (2) types of equipment/modifications, and (3) symptoms across home, school/work, and community settings, and further differentiated between participants with proportionate forms and disproportionate forms of skeletal dysplasia.

**Table 1:** Classification of participants’ clinical diagnoses of a skeletal dysplasia into proportionate or disproportionate conditions, based on the presentation of limb-to-trunk length ratio.

**Other Skeletal Dysplasias**	**Short-limbed Disproportionate Skeletal Dysplasias**
Multiple Epiphyseal Dysplasia	Achondroplasia
Osteogenesis Imperfecta^*^^+^	Cartilage Hair Hypoplasia
Primordial Dwarfism	Hypochondroplasia
Spondyloepiphyseal Dysplasia Congenita^*^	Jeune Syndrome
3 M Syndrome	Pseudoachondroplasia

^*^ *Spondyloepiphyseal Dysplasia Congenita and more severe forms of Osteogenesis Imperfecta present with a disproportionately shorter trunk relative to their limbs.*

*+ Participants with Osteogenesis Imperfecta self-identified as having mild, moderate, or severe presentations.*

## Results

### Participants

A total of 40 participants (12 male) were included in this study. Information regarding age, clinical diagnoses, other sociodemographic characteristics, and comorbidities are presented in Table 2. The STEMS scores, presented in Table 3, demonstrate an increased use of walking aids when ambulating at school/work and in the community, compared with the home setting. Approximately half of the participants experienced both pain and fatigue when mobilizing at school/work (45%) and in the community (57.5%), compared with only 9 (22.5%) participants reporting both symptoms at home.

**Table 2:** Sociodemographic characteristics of participants (*n* = 40).

**Characteristics**		**n (%)**
**Diagnosis**		
Achondroplasia		19 (47.5)
Cartilage Hair Hypoplasia		1 (2.5)
Hypochondroplasia		2 (5)
Jeune Syndrome		1 (2.5)
Multiple Epiphyseal Dysplasia		1 (2.5)
Osteogenesis Imperfecta		8 (20)
Primordial Dwarfism		1 (2.5)
Pseudoachondroplasia		2 (5)
Spondyloepiphyseal Dysplasia Congenita		3 (7.5)
Spondylometaphiphyseal Dysplasia Congenita		1 (2.5)
3 M Syndrome		1 (2.5)
**Gender**		
Female		27 (67.5)
Male		12 (30)
Other		1 (2.5)
**Comorbidity**		
Yes		12 (30)
No		28 (70)
**Occupation** ^*^		
Studying		6 (15)
Working Part-Time		9 (22.5)
Working Full-Time		17 (42.5)
Not Studying/Working		14 (35)
	**Median**	**Range**
Age (years)	41.2	19-68
Height (cm)	128.2	92-155
Body Mass Index	33.4	20.9-54.4

^*^ *Note that the total percentage exceeds 100% as four participants undertook both study and work.*

**Table 3:** Screening Tool for Everyday Mobility and Symptoms scores for participants with skeletal dysplasia (*n* = 40), presented as frequencies and percentages.

	**Home n(%)**	**School/Workn(%)** ^*^	**Community n(%)**
**Walking Aid**			
Independent (score 1)	32 (80)	27 (73)	25 (62.5)
Use of stick (score 2)	3 (7.5)	4 (10.8)	0 (0)
Use of crutches (score 3)	1 (2.5)	0 (0)	3 (7.5)
Use of wheeled walking device (score 4)	1 (2.5)	1 (2.7)	3 (7.5)
Use of wheelchair/mobility scooter (score 5)	3 (7.5)	5 (13.5)	9 (22.5)
**Symptoms**			
Nil pain or fatigue (score A)	23 (57.5)	8 (21.6)	8 (20)
Pain only (score B1)	5 (12.5)	7 (18.9)	5 (12.5)
Fatigue only (score B2)	3 (7.5)	4 (10.8)	4 (10)
Both pain and fatigue (score C)	9 (22.5)	18 (48.6)	23 (57.5)

^*^ *Three participants did not attend school or work, therefore n = 37 within this section.*

A total of 12 participants reported a combined total of 14 comorbidities. The majority of reported comorbidities were neurological or orthopedic, which are the commonly reported associated secondary impairments and comorbidities for individuals with skeletal dysplasias.^2–7^ Comorbidities that were unrelated to individual skeletal dysplasia groups included vestibular migraines, attention-deficit/hyperactivity disorder, depression, and rheumatoid arthritis.

### Functional performance and symptomatology of adults with skeletal dysplasia

**Figure 1:**
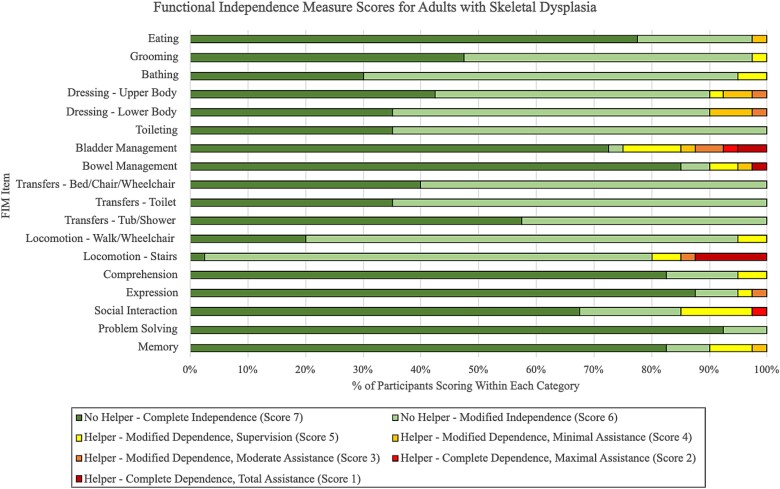
Distribution of functional independence measure (FIM) scores for each item among adults with skeletal dysplasia (*n* = 40).

Participants displayed lower FIM scores across self-care and mobility activities and higher FIM scores within the cognitive domain. There was greater variability in scores among items within the self-care domain when compared with items from the mobility domain (Table 4). The majority of participants scored complete or modified independence across one or more skill areas. Figure 1 represents the distribution of participants scoring within each FIM category for every item.

**Table 4:** Summary statistics of FIM scores for each item, domain subtotals, and FIM total among adults with skeletal dysplasia.

	**Median (FIM score)**	**Range (FIM score)**
**Self-care**		
Eating	7	4-7
Grooming	6	5-7
Bathing	6	5-7
Dressing – Upper Body	6	3-7
Dressing – Lower Body	6	3-7
Toileting	6	6-7
Bladder Management	7	1-7
Bowel Management	7	1-7
Self-care Subtotal (/56)	51	41-56
**Mobility**		
Transfers – Bed/Chair/Wheelchair	6	6-7
Transfers – Toilet	6	6-7
Transfers – Tub/Shower	7	6-7
Locomotion –Walk/Wheelchair	6	5-7
Locomotion – Stairs	6	1-7
Mobility Subtotal (/35)	31.5	24-35
**Cognition**		
Comprehension	7	5-7
Expression	7	3-7
Social Interaction	7	2-7
Problem Solving	7	6-7
Memory	7	4-7
Cognition Subtotal (/35)	34.5	24-35
**FIM Total (/126)**	116	93-126

A summary of the multiple-choice checkbox survey results looking at functional performance and symptomatology for self-care, mobility, and communicative tasks in home, school/work, and community settings is presented in Table 5.

**Table 5:** Summary statistics of checkbox survey questions relating to self-care, mobility, and communication across home, school/work, and community settings, presented as frequencies and percentages (*n* = 40).

	**Home (n/%)**	**School/Work (n/%)**	**Community (n/%)**
**Self-care**			
Assistance from a helper and/or equipment	16 (40)	3 (7.5)	6 (15)
Environmental modifications	21 (52.5)	6 (15)	10 (25)
Experience of pain during or after	7 (17.5)	2 (5)	4 (10)
Experience of fatigue during or after	11 (27.5)	3 (7.5)	5 (12.5)
Experience of other symptoms during or after	6 (15)	1 (2.5)	4 (10)
**Mobility**			
Avoidance of activities	12 (30)	7 (17.5)	27 (67.5)
Environmental modifications	23 (57.5)	13 (32.5)	13 (32.5)
Experience of symptoms during or after	13 (32.5)	12 (30)	22 (55)
**Communication**	**For All Environments**		
Avoidance of activities or environments	7 (17.5)		
Assistance from a helper and/or equipment	4 (10)		
Environmental modifications	2 (5)		
Takes longer compared to average-statured peers	5 (12.5)		

### Self-care

Three (7.5%) participants scored complete independence across all 8 self-care activities. Participants demonstrated highest scores in eating, grooming, bathing, and toileting. Use of bathroom modifications, a step stool, extended reaching tools, shower chair, long-handled brush, anti-slip mats, a dressing stick, and a bidet or wiping device were reported to support independence with these tasks. One (2.5%) participant needed minimal assistance to cut up food due to a strength impairment. Four (10%) participants required assistance from a helper with upper and lower body dressing due to challenges with reaching or because of pain and fatigue. Specific components of dressing tasks that required assistance were doing up zippers and bras, donning/doffing a shirt, putting on orthotics, and accessing clothes from the wardrobe. Strategies reported to improve/increase independence with dressing activities included sitting to dress, avoiding certain clothing items, such as those with back zippers, modifying storage of clothes to improve accessibility, and use of a shoehorn and/or sock aid to assist with lower limb reach. Most participants reported requiring additional time to complete self-care tasks. Bladder and bowel management items scored among the lowest of the 18 items but were reflective of incontinence accidents, rather than the need for physical assistance from a helper. Five (12.5%) participants used pads to manage their incontinence, four (10%) reported taking medication to manage constipation or limit diarrhea, and one (2.5%) participant self-catheterized to ensure complete bladder emptying following a postoperative complication relating to their skeletal dysplasia. Of the 11 participants presenting with urinary urgency or incontinence, 2 reported spinal stenosis as an associated comorbidity.

The survey results revealed that the greatest use of environmental modifications, adaptive equipment, and/or physical assistance from a helper to complete self-care tasks occurred in home settings, with the least modifications or assistance reported across school and/or work environments (Table 5). Some participants reported experiencing pain (17.5%), fatigue (27.5%), and other symptoms (6%) when completing self-care tasks at home. While these symptoms were also reported across school/work and community settings, they were at lower levels than those reported in the home environment (Table 5). Other symptoms reported were shortness of breath, dizziness, pins and needles, stiffness, and numbness.

Four (10%) participants identified bringing a collapsible step stool and portable bidet or perineal hygiene wiping stick with them to school/work or in the community to assist with completion of self-care tasks. Home modifications reported as useful by this population included lower kitchen benches and bathroom sinks, lever taps, adjustable shower heads, walk-in shower, and bathroom rails.

### Mobility

All participants demonstrated complete or modified independence with the 3 transfers items (transfer to chair; transfer to toilet; transfer to bath/shower). Higher levels of independence were observed with tub/shower transfers compared with toilet and bed/chair/wheelchair transfers. Items reported to support independence with transfers included use of a rail, step, electric or lower furniture, and/or holding onto the wall or surroundings for support. Five (12.5%) participants expressed safety concerns when transferring, particularly onto higher or unstable surfaces such as bar stools or wheeled chairs. Most participants reported taking longer to complete transfers compared with peers without a skeletal dysplasia. All participants reported being able to mobilize a minimum of 50 m (which is the required distance for a maximal mobility score); 38 (95%) by walking; and 2 (5%) by wheelchair. One participant required supervision to mobilize this distance. Of the 38 (95%) participants who were ambulant on foot, 6 (15%) reported use of assistive devices (walking stick, crutches, walker, orthotics), and 29 (72.5%) reported taking longer than their average-statured peers. Ascending/descending stairs demonstrated the largest variability in performance within the mobility domain with only 1 (2.5%) participant scoring complete independence (score 7) with stair use. A total of 26 (65%) participants required use of a rail and 18 (45%) reported needing additional time to ambulate a flight of stairs. Four (10%) participants required supervision or physical assistance from a helper, 9 (22.5%) expressed safety concerns with using stairs, and 5 (12.5%) reported avoiding stairs completely. Five (12.5%) participants commented on using an adaptive movement pattern such as crawling, step-to gait, or ambulating backwards on stairs.

Based on survey results, around two-thirds (67.5%) of participants reported avoiding certain activities in at least 1 environment (home, school/work, or community) due to their skeletal dysplasia (Table 5). Across the home environment, activities that were avoided were primarily related to housework and domestic activities of daily living involving reaching and lifting. Two (5%) participants reported avoiding in-person attendance at work and university when possible, preferring to do these activities virtually because of challenges with mobility. Five (12.5%) participants (all with osteogenesis imperfecta) reported avoiding activities and areas that involved a slip or trip hazard, such as mopping, carrying heavy items on stairs, and mobilizing in environments with uneven terrain, slippery surfaces, and/or poor lighting. Participants stated they avoided stairs, walking long distances and prolonged standing, social and sporting activities, grocery shopping, crowds, and places with poor disability access in the community setting.

Over half (52.5%) of participants reported needing adaptive equipment and environmental modifications to their home including lower furniture, step stools, rails, lowered door handles and light switches, and ramps to support access and independence. Across school/work and community environments, participants reported needing a step stool, modified chairs, rails, lifts, wheelchair access, and use of a walking aid or mobility scooter for managing longer distances.

A number of participants reported experiencing symptoms associated with their mobility, more prevalent in the community setting (55%) compared with home (32.5%) and school/work environments (30%) (Table 5). Participants reported symptoms such as pain, fatigue, stiffness, swelling, dizziness, numbness, shortness of breath, pressure areas, and anxiety associated with mobility.

### Cognition

Participants presented with the highest levels of complete independence across the 5 cognitive items, compared with the other domains. The median FIM scores and category subtotal for the 5 cognitive items, comprehension, expression, social interaction, problem solving, and memory, are presented in Table 4, and the percentages of participants scoring within each FIM level are presented in Figure 1. Social interaction demonstrated the lowest levels of complete independence within the cognition domain. Nine (22.5%) participants reported avoiding social situations, seven (17.5%) used anti-anxiety and/or anti-depressant medications, and two (5%) required supervision or prompting in stressful or unfamiliar environments. One (2.5%) participant reported particularly avoiding standing events due to the difficulty interacting with average-statured peers where the height difference is more prevalent, compared with a seated event. Two (5%) participants avoided busy or crowded areas to prevent negative social interactions from others.

Based on the survey results, only a small number of participants required environmental modifications, equipment, assistance from a helper, and/or additional time to support communication (Table 5). In the open-ended text, 1 (2.5%) participant commented that it was difficult communicating to someone behind a high desk and had modified the environment to support direct face-to-face communication instead. Four (10%) participants commented on avoiding busy areas because of difficulty hearing others and being heard when talking, due to their short stature. Symptomatology was not explored with cognitive tasks.

### Comparison of scores between individuals with proportionate and disproportionate forms of skeletal dysplasia

**Figure 2:**
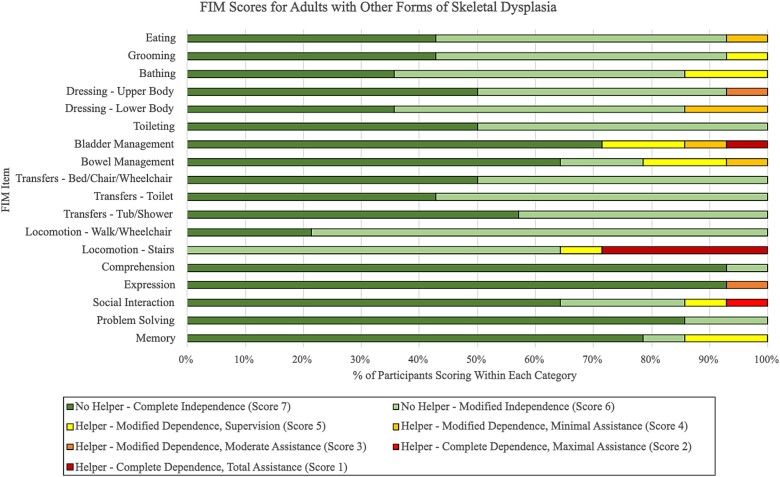
Distribution of functional independence measure (FIM) scores for each item among adults with proportionate forms of skeletal dysplasia (*n* = 14).

**Figure 3:**
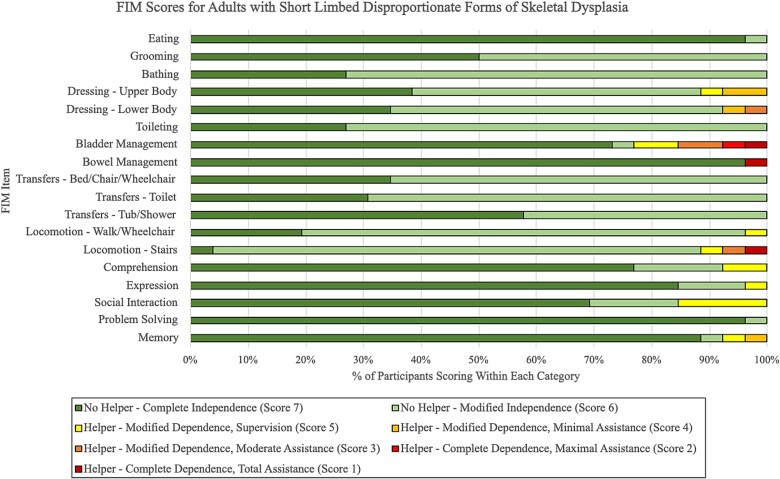
Distribution of functional independence measure (FIM) scores for each item among adults with disproportionate forms of skeletal dysplasia (*n* = 26).

Of the 40 study participants, 14 (35%) were diagnosed with proportionate (median age 36 yr, range 20-67) and 26 (65%) with disproportionate forms (median age 39 yr, range 19-68) of skeletal dysplasia. Within the disproportionate group, all but one participant had conditions presenting with short limbs relative to their trunk, whereas the one participant diagnosed with spondyloepiphyseal dysplasia congenita presented with a short trunk relative to their limbs. Based on the median FIM scores, participants within the proportionate group demonstrated higher levels of independence in upper body dressing, toileting, and bed/chair/wheelchair transfers, whereas the participants in the disproportionate group had higher scores in eating and grooming tasks (Table 6). Median FIM scores for all other individual items were equal between both groups, and both groups demonstrated generally similar overall ranges (Table 6). The proportionate group had slightly lower median subtotal scores for self-care and cognition, but a higher median total FIM score, compared with the disproportionate group (Table 6). One participant in the disproportionate group was completely independent with all activities, scoring 126/126. The distribution of proportionate and disproportionate participants scoring within each FIM grading level for every item are displayed in Figures 2 and 3, respectively.

**Table 6:** Summary statistics of FIM scores for each item, domain subtotals, and FIM total among adults with proportionate and disproportionate forms of skeletal dysplasia.

	**Other Skeletal Dysplasias (n = 14)**		**Short-limbed Disproportionate Skeletal Dysplasias (n = 26)**	
	**Median**	**Range**	**Median**	**Range**
**Self-care**				
Eating	6	4-7	7	4-7
Grooming	6	5-7	6.5	5-7
Bathing	6	5-7	6	5-7
Dressing – Upper Body	6.5	3-7	6	3-7
Dressing – Lower Body	6	4-7	6	3-7
Toileting	6.5	6-7	6	6-7
Bladder Management	7	1-7	7	1-7
Bowel Management	7	4-7	7	1-7
Self-care Subtotal (/35)	50.5	41-56	52	41-56
**Mobility**				
Transfers – Bed/Chair/Wheelchair	6.5	6-7	6	6-7
Transfers – Toilet	6	6-7	6	6-7
Transfers – Tub/Shower	7	6-7	7	6-7
Locomotion – Walk/Wheelchair	6	5-7	6	5-7
Locomotion – Stairs	6	1-7	6	1-7
Mobility Subtotal (/35)	32	24-34	31	24-35
**Cognition**				
Comprehension	7	6-7	7	5-7
Expression	7	3-7	7	3-7
Social Interaction	7	2-7	7	2-7
Problem Solving	7	6-7	7	6-7
Memory	7	5-7	7	4-7
Cognition Subtotal (/35)	34	24-35	35	24-35
**FIM Total (/126)**	116.5	93-125	116	93-126

## Discussion

To our knowledge, this is the first study to objectively measure functional performance in a general skeletal dysplasia population, and to describe differences between individuals with proportionate and disproportionate forms of skeletal dysplasia. This research supports previous findings by providing quantitative and qualitative data on challenges associated with functional independence across everyday activities for adults with skeletal dysplasia.^5^^,^^9^^,^^10^^,^^15^^,^^24^ While healthy and functional adults typically achieve complete independence across all 18 skill areas on the FIM (score 126 out of 126), this was only achieved by 1 participant within our cohort, and the median score was 10 points lower than what is maximally attainable on the FIM across the group. Overall, participants required the greatest levels of assistance within the self-care and mobility domains.

This study provides qualitative and quantitative data regarding physical function across a broad range of 18 self-care tasks for individuals with skeletal dysplasia. Previous research has focused on individual activities such as toileting, dressing, and bathing.^4^^,^^9^^,^^10^ Our findings revealed self-care tasks to be relatively challenging for this population, and the lower FIM scores reported among this cohort are likely a result of the clinical presentation of skeletal dysplasia. Most participants completed self-care tasks independently, which is consistent with Alade et al’s^10^ findings. However, participants in this study reported a high reliance on assistive equipment usage or increased time (score 6—modified independence) to successfully complete self-care activities. Adaptive equipment reported as useful was the one that supported access and reach, which address both the primary impairments of short stature and commonly disproportionate limbs, respectively, for this population. Surprisingly, bladder and bowel management revealed the greatest range of scores, with several participants reporting incontinence accidents secondary to spinal stenosis.^3^^,^^4^ Fatigue was the most common symptom experienced during self-care activities, followed by pain, then other symptoms. Overall, the increased time required by most participants to complete self-care activities may be a result of using assistive equipment, experiencing symptoms, or using adaptive physical strategies, such as sitting to dress or shower, to complete tasks.

Reduced stature may create physical challenges with mobility, when navigating an environment designed for individuals of average-stature. This study found the majority of participants used equipment or environmental modifications to support access and transfers across environments. Individuals with skeletal dysplasia demonstrate a shorter stride length when mobilizing due to the shortened length of their lower limbs, and therefore may exhibit either slower walking speeds or increased cadence, requiring an increase in energy expenditure, to match an equivalent speed of their average-statured peers.^4^ This is supported by our findings, as most participants scoring 6 (modified independence) with walking reported taking longer to complete this task. Furthermore, the altered biomechanics of extreme short stature may directly impact the strength and ranges of motion required to ascend and descend a flight of standard height stairs. Several participants reported safety concerns when using stairs, and most participants reported using the handrail when using stairs. Consistent with reports in the literature,^4^^,^^5^^,^^8^^,^^9^^,^^12^ symptoms of pain, fatigue, and other symptoms associated with relevant comorbidities were frequently reported within this cohort, which is also likely to impact motor performance. Symptoms were more prevalent across community settings, where increased walking distances are generally required, and the surroundings may not be optimized or modified for this population compared with home and school/work environments.

Cognitive tasks on the FIM were identified as a relative strength among participants. This is not unexpected, as the presenting conditions in this cohort are not typically known to have intellectual impairments in comparison with other forms of skeletal dysplasia.^7^ However, participants demonstrated lower scores across the social interaction item, due to a conscious avoidance of social settings, and reported anxiety and depression associated with these. Participants specifically commented on difficulties communicating with their peers due to height differences, as well as avoidance of populous environments to minimize negative interactions and judgment from others. This study identified challenges for this population when interacting with others of average-stature, although further qualitative assessment of this is needed to explore this further and better understand the social impacts associated with living with a skeletal dysplasia.

It is anticipated that individuals with disproportionate conditions would experience more physical challenges with reaching activities due to the biomechanics of their atypical trunk-to-limb length ratio, which has been found to be the strongest predictor of poorer performance in self-care activities.^5^ Toileting, dressing, and bathing all involve a significant reach component and have been reported to be particularly difficult to perform among this population group,^4^^,^^9^^,^^10^ This is consistent with findings from this study, with a greater use of equipment and modifications for toileting, dressing, and bathing reported by participants with disproportionate stature, compared with those in the proportionate group.

There is little existing research examining physical functioning in individuals with proportionate forms of skeletal dysplasia. Apart from toileting, dressing, and bathing, this study found FIM scores (medians and ranges) across all other items to be similar between proportionate and disproportionate individuals. This suggests that the functional challenges experienced by adults with skeletal dysplasia are more related to their reduced stature, a common anatomical trait shared between the two groups. However, individuals with osteogenesis imperfecta (proportionate) specifically reported more safety concerns associated with mobility and eating likely associated with bone and teeth fragility, which are impairments relevant to this diagnosis and not commonly seen with other forms of skeletal dysplasia.^25^

The main limitation of this study was the risk of self-selection bias as participants were recruited through advocacy groups. Our cohort consisted of a small sample size; however, this is not unusual when recruiting a population with rare genetic disorders. Another limitation was that qualifying comments were not made by assessors on all FIM assessments, meaning that conclusions could not be drawn on why some participants received a certain score. This study utilized a standardized validated instrument, the FIM, that provided quantitative data on functional performance. There was variety in the age and diagnoses among the participants, thus providing an increased understanding of the functional impact of skeletal dysplasias more generally as a whole. The unequal numbers of participants with proportionate and disproportionate conditions were representative of the greater population, as achondroplasia, a disproportionate growth disorder, is the most prevalent form of skeletal dysplasia.^3^

This study provides a snapshot of the levels of functional independence and symptomatology associated with this, among adults with skeletal dysplasia. It also provided insight regarding the types of equipment and modifications that are effective at improving independence for this population group. Future research should consider a more thorough qualitative analysis of what barriers may exist in accessing those assistive supports. A broader recruitment method is suggested to reduce risk of selection bias and increase chances of recruiting more participants, which may allow for stronger statistical analyses to be performed. The data collected in this study may serve as a baseline for future longitudinal studies, to evaluate differences in FIM score after an intervention has been introduced.

## Conclusion

Adults with skeletal dysplasia demonstrate lower functional independence scores in everyday self-care, mobility, and cognitive activities. Individuals with disproportionate forms of skeletal dysplasia may present with additional challenges in self-care activities that require reaching, as well as bed/chair/wheelchair and toilet transfers, compared with their peers with proportionate conditions. Use of equipment, modifications, and allowing additional time to complete tasks are effective strategies to increase levels of functional independence. Prospective research is needed to further evaluate what assistive devices or therapies are useful and what potential barriers may exist in accessing these forms of support among this population group.

## Data Availability

The data sets used within this study are stored securely and may be accessed upon reasonable request to the author(s).
